# **Single trabecular titanium Cup-Cage for acetabular bone defects during revision hip arthroplasty: Mid-term outcomes**

**DOI:** 10.1186/s13018-025-06553-x

**Published:** 2025-12-13

**Authors:** Mahmoud Fahmy, Mahmoud Abdel Karim, Mohamed Abo-Elsoud, Mostafa Ahmed Shawky

**Affiliations:** Arthroplasty and Pelvis fractures Unit, Orthopaedic Department, Kasr Alainy Hospital, Cairo University, Cairo, Egypt

**Keywords:** Revision hip arthroplasty, Trabecular titanium, Acetabular bone defects, Delta TT system, Cup-cage construct

## Abstract

**Background:**

Severe acetabular bone loss remains a major challenge in revision total hip arthroplasty (rTHA). Traditional cup-cage techniques can provide stability but are associated with technical complexity and mixed long-term outcomes. The Delta TT system, a trabecular titanium construct functioning as a single cementless cup-cage, may combine biological fixation with mechanical reliability. This study aimed to evaluate its mid-term clinical and radiographic outcomes in complex acetabular reconstructions.

**Methods:**

In this prospective cohort (2018–2021), 64 patients (mean age: 68.4 years) with Paprosky type II (*n* = 38) or type III (*n* = 26) acetabular defects underwent rTHA using the Delta TT system. Functional outcomes were measured with the Merle d’Aubigné and Postel score preoperatively and at a mean 48-month follow-up. Radiographs were assessed for implant stability, osteointegration, and radiolucency. Complications and re-revision rates were documented.

**Results:**

Stable radiographic fixation was achieved in nearly all cases. Two patients demonstrated minor, non-progressive radiolucency without clinical significance. Osteointegration was evident in all hips within 12 months. Functional outcomes improved significantly, with mean scores increasing from 8.2 preoperatively to 15.6 at final follow-up (*p* < 0.001). At mid-term, 75% of patients achieved good-to-excellent clinical results. The complication rate was low, and no re-revisions were required for aseptic loosening.

**Conclusion:**

The Delta TT system offers a reliable and biologically favorable solution for managing severe acetabular defects in rTHA. Its porous trabecular titanium structure and modular design promote durable fixation, radiographic stability, and significant functional recovery, with low complication rates. These mid-term results support its role as a valuable reconstructive option in complex revision hip arthroplasty. Long-term and comparative studies are warranted to further validate durability and outcomes.

**Supplementary Information:**

The online version contains supplementary material available at 10.1186/s13018-025-06553-x.

## Introduction

Revision total hip arthroplasty (rTHA) has become an increasingly common and complex procedure as the number of primary total hip replacements continues to rise worldwide [1–5]. One of the most significant challenges encountered during rTHA is the management of severe acetabular bone loss, which can result from factors such as aseptic loosening, osteolysis, periprosthetic joint infection, or component migration [5–10]. These defects can significantly compromise the ability to achieve stable implant fixation and restore normal hip biomechanics, often necessitating advanced reconstructive techniques [10–15].

In cases of severe bone loss, restoration of the acetabular bone stock, center of rotation, and mechanical stability is critical but technically demanding [16–20]. The most common reconstructive option is the use of modular, off-the-shelf porous acetabular components and augments, which allow reliable biological fixation [21–25]. However, their implantation requires advanced surgical expertise, including precise fitting of the modular components and unitization of the construct with cement, which may increase the technical complexity of the procedure. Traditional alternatives include structural allografts, anti-protrusio cages, or custom triflange components. However, these methods have shown variable long-term success, with complications including graft resorption, mechanical failure, and limited biological fixation [24–29].

In recent years, porous metal implants have emerged as a promising alternative for managing complex acetabular defects. The Delta TT (Trabecular Titanium) acetabular system represents a new generation of modular revision systems designed specifically to address severe bone loss [7]. It provides a highly porous, biomimetic structure that closely resembles natural trabecular bone. This unique architecture facilitates rapid bone ingrowth and long-term osseointegration [8, 9], while its modularity allows for intraoperative flexibility in restoring the hip center and achieving stable fixation even in cases with minimal host bone. Importantly, the system enables the creation of an ‘off-the-shelf’ flange construct that offers similar advantages to patient-specific custom designs, but without the need for preoperative planning and manufacturing delays [10–18].

The purpose of this study is to evaluate the clinical and radiographic outcomes associated with the use of the Delta TT acetabular system in the treatment of severe acetabular bone defects during revision hip arthroplasty. We aim to assess implant survival, complication rates, functional outcomes, and the radiographic evidence of osseointegration to provide further insight into the role of these implants in complex revision scenarios and to clarify their potential advantages in promoting durable and biologically active acetabular reconstruction.

## Patient and methods

From June 2018 to January 2021, a prospective trial was conducted at a university hospital after approval from the institutional ethics committee (N-98-2025). Patients undergoing revision total hip arthroplasty (rTHA) for failed acetabular components due to aseptic or septic loosening, polyethylene wear, osteolysis, recurrent dislocation, periprosthetic fracture, or failure after hemiarthroplasty were included. Exclusion criteria comprised neglected acetabular fractures untreated within three weeks, pathological acetabular fractures, or unreconstructable acetabular fractures requiring arthroplasty. Patients with prior infection underwent a two-stage revision: initial implant removal, thorough debridement, and placement of an antibiotic-loaded cement spacer, followed by reimplantation after infection control.

All patients received preoperative clinical and radiological assessments, including plain radiographs and CT scans. All patients provided written informed consent prior to their enrollment. Acetabular defects were classified using the Paprosky system (mostly type II, some type III). Demographic data, failure cause, and defect classification were recorded. All procedures were performed by a senior arthroplasty consultant from the authors.

Surgery was performed in the lateral position via a posterior approach under spinal-epidural anesthesia. Acetabular components were carefully removed to minimize bone loss, with intraoperative reassessment to confirm reconstruction planning. The femoral stem was revised if loosening was detected. Extended trochanteric osteotomy (ETO) was performed when required, creating a controlled longitudinal cortical window along the lateral femoral cortex; the fragment was reattached and stabilized with cerclage wires after stem removal and canal preparation.

**Fig. 1:**
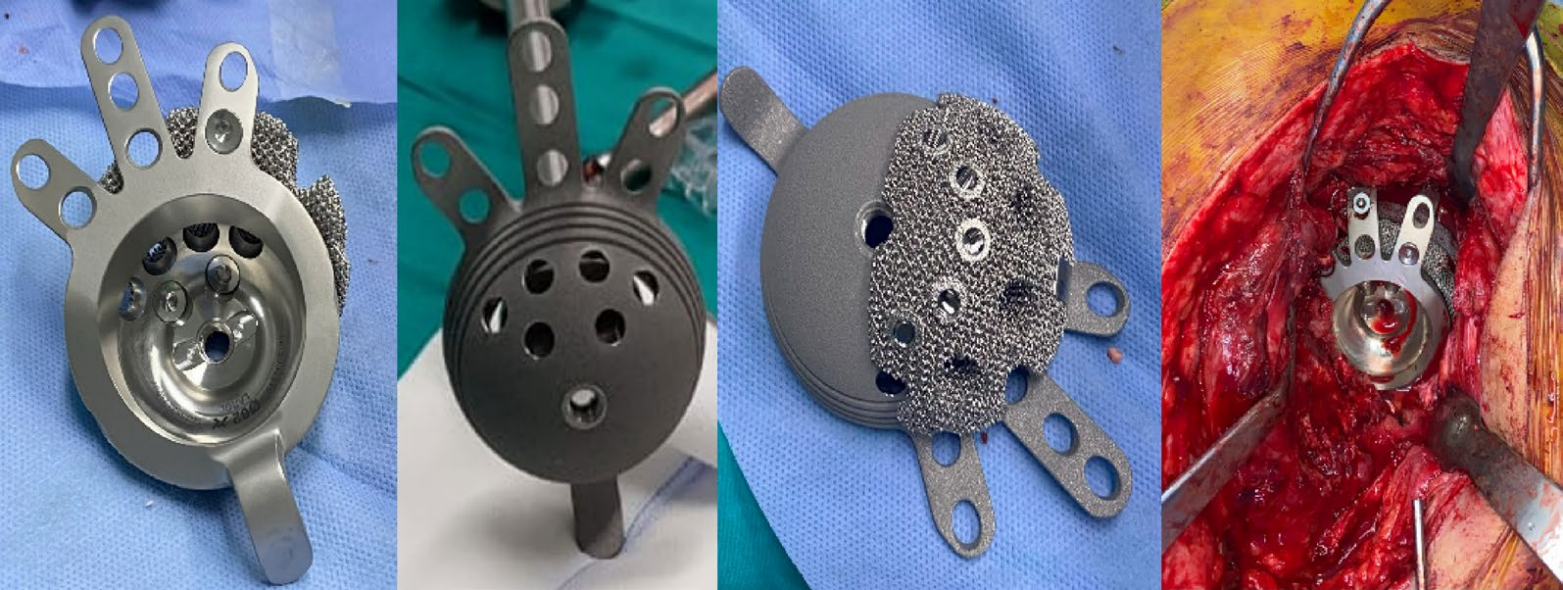
Intraoperative photo showing cementless-cup cage with possible application of trabecular titanium at its upper surface with screws (no cement) before and after implantation

The Delta TT revision system (LimaCorporate, Udine, Italy) was used in all cases. This cementless cup–cage construct provides distal ischial and iliac fixation for stability. Porous titanium augments were used for segmental bone loss, and internal spacers corrected cup orientation or medial cavitary defects (Fig. 1). Morselized allogenic bone grafts were impacted when bone stock was compromised. Postoperatively, all patients, including those who underwent ETO, generally followed a rehabilitation program of partial weight-bearing ambulation for six weeks, with progression to full weight-bearing once radiological evidence of osteointegration was confirmed, allowing adjustments according to individual case scenarios.

Radiographically, patients were evaluated monthly using postoperative radiographs to assess radiolucency using DeLee and Charnley’s classification [9], cup migration using Harris-Galante and Charnley criteria [10], osteointegration progression, and screw integrity.

Functionally, patients were evaluated monthly using the Merle d’Aubigné and Postel score until final follow-up (mean 48 months). Any postoperative complications such as infection or dislocation, periprosthetic fractures were documented.

Statistical Analysis: Continuous data are presented as mean ± SD. Pre- and postoperative Merle d’Aubigné scores were compared using the Wilcoxon signed-rank test; group comparisons were performed with the Mann–Whitney U or Kruskal–Wallis tests as appropriate. A p-value < 0.05 was considered significant. Analyses were conducted using SPSS version 26 (IBM Corp., Armonk, NY, USA).

## Results

**Fig. 2:**
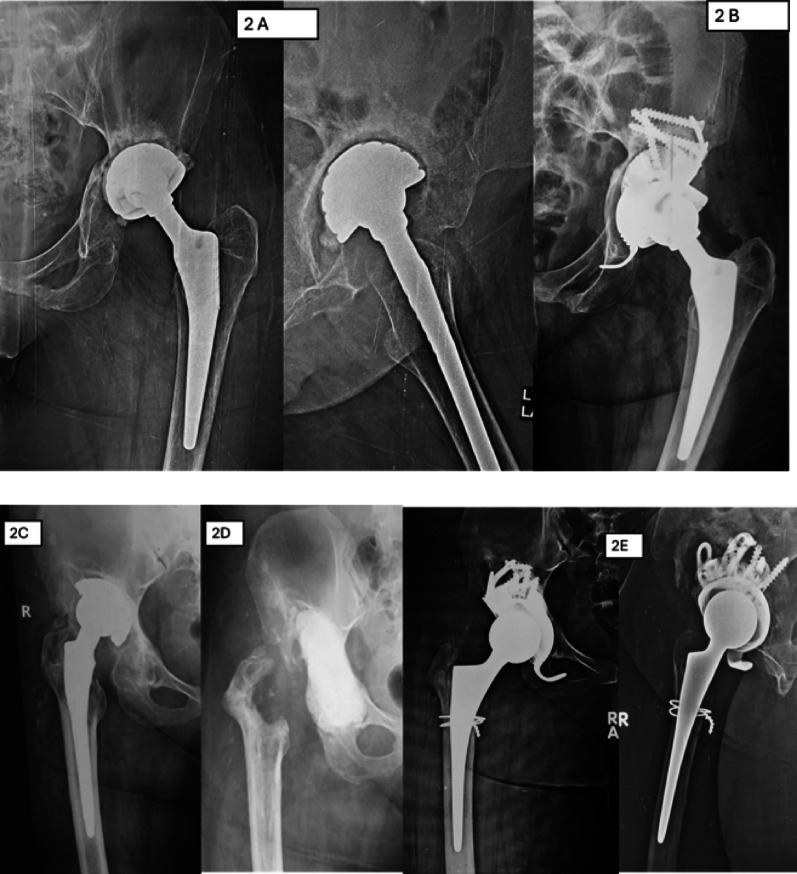
Case examples of Type III Paparosky management: **A** Preoperative radiograph showing failed hemiarthroplasty. **B** Postoperative radiograph at the final follow up visit after Delta TT system implantation. **C** Preoperative radiograph showing septic loosening of cementless acetabular cup. **D** Preoperative radiograph showing implant removal. **E** Postoperative radiograph at the final follow up visit after Delta TT system implantation

**Fig. 3:**
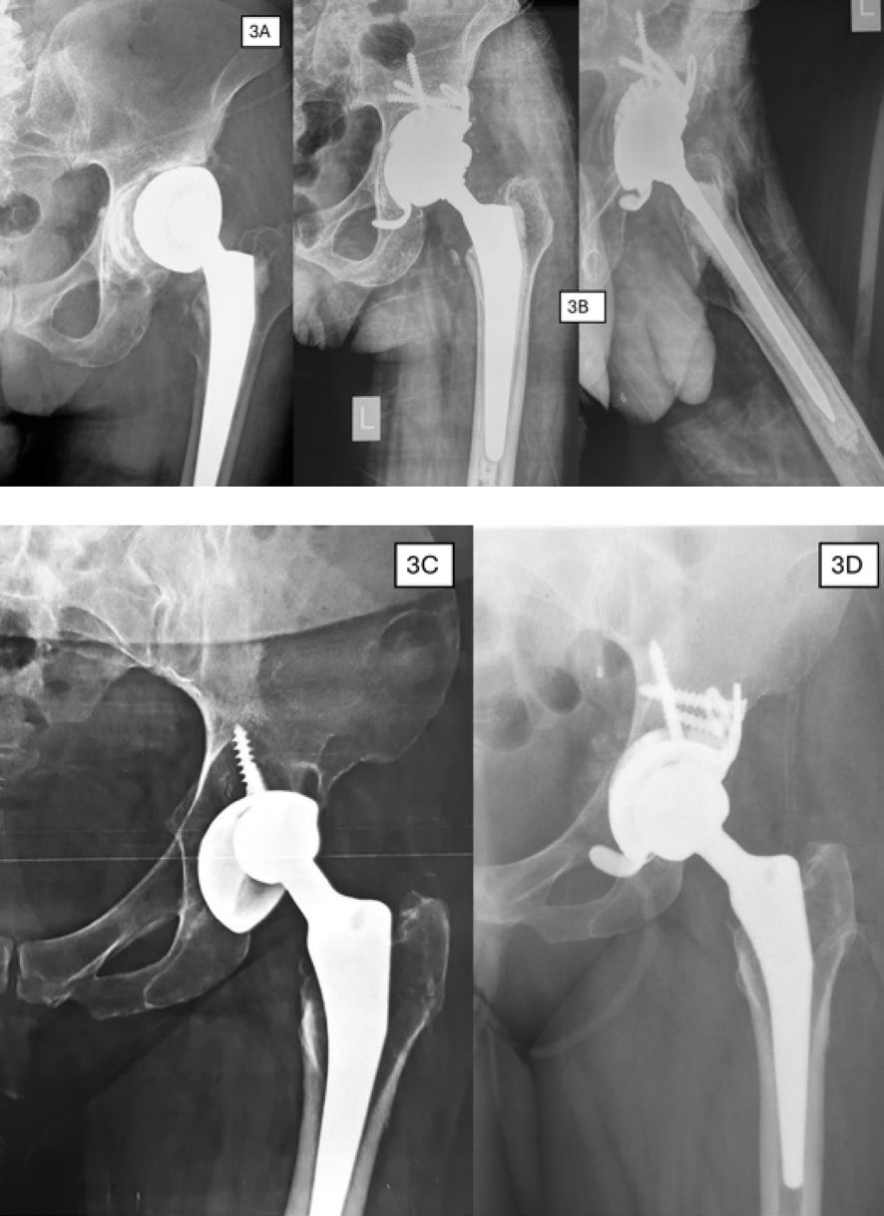
Case examples of Type II Paparosky management: **A** Preoperative radiograph showing aseptic loosening of cemented dual mobility cup. **B** Postoperative radiograph at the final follow up visit after Delta TT system implantation. **C** Preoperative radiograph showing aseptic loosening of cementless acetabular cup. **D** Postoperative radiograph at the final follow up visit after Delta TT system implantation

During the study period, the Delta TT system was used in 70 cases; 6 were excluded because they involved neglected complex acetabular fractures treated as primary surgeries rather than revisions. The final cohort comprised 64 patients (36 males, 28 females) with a mean age of 68.4 ± 7.8 years (range 50–82) and a mean follow-up of 48 ± 6 months (range 44–60). Preoperative Paprosky classification showed 38 Type II defects (IIA: 15, IIB: 13, IIC: 10) and 26 Type III defects (IIIA: 18, IIIB: 8). Revision indications included aseptic loosening (48 patients, 75%), septic loosening (11, 17%), metallosis/wear (3, 4.7%), and dislocation (2, 3.1%) [Table 1] [Figs. 2 and 3 as case examples]. A trabecular titanium augment was in 32 cases, while internal central spacers were utilized in 30 patients. Bone grafting was performed in three patients (4.7%). Extended trochanteric osteotomy was performed in 10 cases for central prosthesis dislocation and in 5 cases for stem removal .

**Table 1:** Demographic and preoperative characteristics of the study cohort

Parameter	Value
Number of patients	64
Sex (Male/Female)	36/28
Mean age (years)	68.4 ± 7.8 (50–82)
Mean follow-up duration (months)	48 ± 6 (44–60)
Operated side	Right = 34 hips/Left = 30 hips
Paprosky classification	
Type IIA	15 (23.4%)
Type IIB	13 (20.3%)
Type IIC	10 (15.6%)
Type IIIA	18 (28.1%)
Type IIIB	8 (12.5%)
Indication for revision (Cause of failure)	
Aseptic loosening	48 (75%)
Septic loosening	11 (17.2%)
Metallosis/Wear	3 (4.7%)
Recurrent dislocation	2 (3.1%)
Reconstructive adjuncts used	
Porous titanium augment	32 (50%)
Internal central spacer	30 (46.9%)
Bone graft	3 (4.7%)
Extended trochanteric osteotomy (ETO)	15 (23.4%) 10 for central dislocation, 5 for stem removal

Radiological and functional outcomes are summarized in Table 2. At the final follow-up, radiolucent lines were observed in 9 patients (14%), all < 2 mm and non-progressive. Two patients (3.1%) showed non-progressive cup migration < 2 mm. No complete three-zone radiolucency or implant migration > 2 mm was noted. Functionally, Merle d’Aubigné and Postel scores improved significantly from 8.2 ± 1.4 preoperatively to 15.6 ± 1.8 at final follow-up (*p* < 0.001; Δ = 7.4 ± 2.1; 95% CI 6.8–8.0) with good to excellent outcomes (≥ 15) achieved in 48 patients (75%). Subgroup analysis is summarized in Table 2.

**Table 2:** Functional, Radiological, and complication outcomes

Parameter	Preoperative	Postoperative (Final Follow-up)
Functional outcome		
Merle d’Aubigné & Postel score (mean ± SD)	8.2 ± 1.4	15.6 ± 1.8 (*p* < 0.001; Δ = 7.4 ± 2.1; 95% CI 6.8–8.0)
Good to excellent (≥ 15)	0 (0%)	48 (75%)
Fair (13–14)	3 (4.7%)	14 (21.9%)
Poor (< 13)	61 (95.3%)	2 (3.1%)
Radiological outcome		
Radiolucent lines (> 2 mm or progressive)	–	9 (14%) – Zone I (7), Zone II (2)
Cup migration (> 1 mm)	–	2 (3.1%) < 2 mm, non-progressive
Osteointegration signs	–	Grafted cases showed graft incorporation within 9 months with stable fixation, while non-grafted cases achieved radiographic osteointegration by 12 months with no significant difference in function (*p* = 0.62; 95% CI − 1.5 to 2.4).
Screw failure	–	1 (1.6%) asymptomatic
Time to union for ETO		All patients with ETO achieved union at a mean of 4.2 ± 0.6 months.
Subgroup analysis		
Functional outcomes between Paprosky II and III defects	ــ	No significant difference between Paprosky II and III defects (15.8 ± 1.7 vs. 15.4 ± 1.9; *p* = 0.37; 95% CI − 0.5 to 1.3)
Functional outcomes between ETO vs. non-ETO groups	ــ	Final functional scores were comparable between ETO and non-ETO groups (15.3 ± 1.6 vs. 15.7 ± 1.8; *p* = 0.49; 95% CI − 0.7 to 1.4).
Complications		
Superficial surgical site infection	–	2 (3.1%)
Deep infection	–	1 (1.6%) — managed with DAIR, no recurrence
Dislocation	–	0 (0%)
Aseptic loosening	48 (75%) (cause of revision)	0 (0%)
Kaplan–Meier survival	–	98.4% (95% CI 96.3–100) for any cause; 100% for aseptic loosening

Postoperative complications (Table 2) occurred in 3 patients (4.7%; 95% CI 1.0–13.1), including 2 superficial infections (3.1%) successfully treated with antibiotics and 1 deep periprosthetic infection (1.6%) managed with a DAIR (Debridement, Antibiotics, and Implant Retention) protocol followed by six weeks of intravenous culture-specific antibiotics and an additional six weeks of oral suppressive therapy under infectious-diseases supervision. Implant retention was achieved with no recurrence.

Radiographic and functional outcomes remained excellent throughout follow-up. No cases of dislocation, aseptic loosening, or periprosthetic fracture were detected. Kaplan–Meier survival at a mean of 48 months was 98.4% (95% CI 96.3–100) for revision due to any cause and 100% for aseptic loosening. Radiographic stability, osteointegration, and functional recovery were both statistically and clinically significant.

## Discussion

 Major acetabular bone loss in revision total hip arthroplasty remains difficult due to severe defects, distorted anatomy, and deficient bone stock, while traditional options, bulk allografts, cages, or triflange implants have clear limitations [3, 4, 6, 19–25]. Modular, high-porosity trabecular titanium (Delta TT) components offer biomimetic architecture promoting osseointegration, durable fixation, and intraoperative flexibility using augments, spacers, or offset liners [5, 7, 8, 11–18]. Multipoint fixation with screws or iliac/ischial extensions enhances stability and restores hip center in Paprosky II–IIIdefects [8, 12–22]. Combining mechanical stability and biological fixation, Delta TT provides a reliable solution for complex reconstructions [11, 12, 23, 24].

 In our series, the Delta TT system demonstrated favorable clinical and radiographic outcomes at a mean follow-up of 48 months. Postoperative complications occurred in only three patients (4.7%): two superficial surgical site infections, successfully managed with oral antibiotics and local wound care, and one deep infection treated with surgical debridement and prolonged antibiotic therapy with implant retention. Despite a seemingly higher infection rate, our results consistent with the 3–6% infection rates described in similar complex rTHA cohorts [12, 13, 15].No dislocations, aseptic loosening, periprosthetic fractures, or hardware failures were observed, indicating excellent mechanical stability of the construct. These results suggest that Delta TT reconstructions can minimize mechanical complications while providing reliable fixation, even in severe bone loss.

 Comparison with published series further contextualizes our findings. Cacciola et al., in a multicenter cohort of 102 Delta TT revisions (Paprosky II–III), reported rare mechanical failures (~ 1–2%), periprosthetic fractures (< 2%), and infections (~ 3%) over 48 months, with consistent functional improvements measured by HHS [14]. Our cohort showed a slightly higher overall complication rate (4.7%), but importantly, all mechanical complications were absent, and functional recovery (Merle d’Aubigné 15.6 ± 1.8) was comparable. Munegato et al., analyzing 78 Paprosky III defects, observed no re-revisions for loosening or infection and stable cup integration at 39.8 months [12], aligning with our zero cases of aseptic loosening or migration >2 mm. El Ghazawy et al., in 42 Delta TT revisions without routine structural allografts, reported minimal migration, progressive osseointegration, and durable fixation [13], corroborating our observations of complete osteointegration by 12 months and absence of mechanical failure.

 Perticarini et al., in 125 acetabular revisions, observed low loosening rates (~ 2%), minimal complications, and consistent functional improvement [11], while Puig-Ruano et al. focused on Paprosky IIIB defects and demonstrated excellent hip center restoration, optimal load transfer, and significant functional gains [15]. Steno et al. emphasized the role of iliac and ischial screw fixation in reducing micromotion and early migration in pelvic discontinuities [17], consistent with our zero mechanical failures, likely aided by multipoint fixation. De Meo et al. reported mid-term success in 65 cases using modular augments with limited structural grafting [8], and Vasios et al., in 28 complex aseptic loosening cases, confirmed reproducible fixation, minimal migration, and favorable functional outcomes [18]. Across these series, dislocation rates ranged from 0 to 7%, aseptic loosening 0–2%, and periprosthetic fractures 0–2%, highlighting that our cohort’s absence of these complications further reinforces the Delta TT system’s mechanical reliability [7–18].

 Overall, our series corroborates prior evidence that Delta TT reconstructions provide durable fixation, low rates of major complications, and significant functional improvement. While infection remains the most common complication, the majority were superficial and managed successfully. The absence of dislocations, aseptic loosening, or hardware failures in our cohort, combined with consistent functional gains (75% good-to-excellent Merle d’Aubigné scores), demonstrates outcomes comparable to or slightly better than previously published multicenter and single-center series. Radiographic follow-up across these Delta TT series consistently demonstrates high implant stability, progressive osseointegration, and minimal migration, with follow-up durations ranging from 36 to 60 months [7, 11–15, 17, 18]. A comparative evaluation of our results with existing literature demonstrates that modular augments with multipoint screw fixation achieve consistent mid-term survivorship and functional outcomes across different Paprosky classifications, confirming the Delta TT system’s dependable performance in complex acetabular reconstruction. [7–18].

On the other hand, conventional cages and jumbo cups are associated with higher rates of loosening, instability, and infection in severe defects [3, 6]. Our findings, in line with previous reports, indicate that modular, off-the-shelf systems like Delta TT provide comparable stability with lower operative complexity and faster rehabilitation [11, 12].

Structural allografts offer biological scaffolding and potential long-term integration [19, 20, 23], but outcomes are often limited by resorption and mechanical failure [19, 20, 23]. Delta TT with modular augments and multipoint fixation reduces reliance on bulk grafts, adapting to variable defects while minimizing early mechanical failure [13–15].

Cup-cage constructs achieve robust fixation but require extensive exposure, longer surgery, and may increase infection risk [21, 24]. Their rigidity limits intraoperative adaptability, whereas Delta TT allows real-time customization, maintaining stability and function while reducing operative burden [12–15].

Triflange implants suit extreme defects but are costly, time-consuming, and may not fit as planned [22, 25]. Delta TT provides off-the-shelf adaptability for most Paprosky II–III defects [13–15, 18].

Modular porous systems combine stability, biological fixation, and intraoperative flexibility, enabling less invasive surgery, earlier mobilization, and consistent restoration of hip mechanics. The Delta TT system is particularly suitable for Paprosky II–III defects where its modular design allows real-time adaptation to defect morphology without extensive grafting or custom implants, and off-the-shelf availability reduces preoperative planning time. Modular augments and adjustable offsets restore the hip center and optimize load transfer. In our series, these features resulted in comparable complication rates, minimal dislocations, and sustained functional outcomes, consistent with prior studies [12–18].

While prior studies have reported favorable outcomes with the Delta TT system [11–15], our study contributes additional insights. It represents one of the longest mid-term follow-up series (mean 48 months), offering information on durability and biological performance in complex revisions. It also includes a heterogeneous population of Paprosky II and III defects, reflecting real-world challenges and enhancing generalizability. Compared with prior Delta TT series, our cohort includes a broader range of Paprosky II–III defects and demonstrates comparable mid-term survivorship. Furthermore, we analyze modular augment utilization, multipoint fixation, and hip center restoration, directly linking surgical technique to radiographic and functional outcomes. Finally, practical intraoperative considerations demonstrate how modularity can reduce reliance on cages or patient-specific implants while maintaining biomechanical stability.

Limitations of this study include the absence of a direct comparative control group, the single-center design, and potential surgeon selection bias. Although the mean follow-up is substantial, longer-term evaluation (≥ 10 years) will be required to fully assess implant durability and late complications. Future prospective, multicenter, randomized studies comparing Delta TT with bulk grafting, modular cup-cages, and patient-specific triflange implants are warranted to provide high-level evidence guiding implant selection in complex rTHA.

## Conclusion

The Delta TT system provides excellent mid-term outcomes in revision total hip arthroplasty. Radiologically, stable osteointegration was achieved in most cases, with minimal radiolucent lines and no implant migration or loosening at a mean follow-up of 48 months. Functionally, patients demonstrated significant improvements in clinical scores, reflecting effective pain relief, mobility, and ambulation. Postoperative complications included two cases of superficial surgical site infection and one deep infection, all managed successfully. These findings support the clinical effectiveness and reliability of the Delta TT system for managing complex acetabular bone defects during revision procedures.

## Supplementary Information

Below is the link to the electronic supplementary material.

Supplementary Material 1

## Data Availability

The datasets used and/or analyzed during the current study available from the corresponding author on reasonable request.
